# Silent sinus syndrome: A case report

**DOI:** 10.1002/ccr3.8095

**Published:** 2023-10-25

**Authors:** Samaneh Salari, Najme Anbiaee, Maryam Bozorgi Baee

**Affiliations:** ^1^ Oral and Maxillofacial Medicine Oral and Maxillofacial Diseases Research Center, Mashhad University of Medical Sciences Mashhad Iran; ^2^ Oral and Maxillofacial Radiology Oral and Maxillofacial Diseases Research Center, Mashhad University of Medical Sciences Mashhad Iran; ^3^ Oral and Maxillofacial Medicine Mashhad University of Medical Sciences Mashhad Iran

**Keywords:** atelectasia, enophthalmos, maxillary sinus, silent sinus syndrome

## Abstract

A 65‐year‐old patient was referred to the Faculty of Dentistry, Mashhad University of Medical Sciences, Mashhad, Iran, with left facial pain and numbness in the upper lip. Based on clinical examinations and radiographic investigations, the patient was diagnosed with silent sinus syndrome. This study discusses oral findings associated with silent sinus syndrome.

## 1 INTRODUCTION

Silent sinus syndrome was first defined by Montgomery in 1964, and 30 years later, Soparker et al. used the term “silent sinus syndrome” to describe enophthalmos with Atelectasia.* ^1^, ^2^

This syndrome is caused by the negative pressure of the maxillary sinus due to the obstruction of the infundibulum, which results in the maxillary sinus walls gradually contracting and pulling inward, as well as the orbital floor retracting downward. The retraction of the orbital floor downward can cause hypoglobus.† ^3^, ^4^

Silent sinus syndrome occurs mostly in the third to fifth decades of life. The relationship between gender, profession, smoking, alcohol, heredity, and silent sinus syndrome has not been confirmed. However, idiopathic, iatrogenic, and trauma factors have been suggested in its development.^3^, ^5^ The clinical symptoms of silent sinus syndrome include Blepharoptosis,‡ enophthalmia,§ and, in rare cases, diplopia, and ptosis.^3^, ^6^ Although this syndrome can be initially diagnosed clinically, it must be confirmed through radiology.^7^

So far, a small number of these patients have been reported, the majority of whom were diagnosed by radiologists and ophthalmologists. Since the quick and correct diagnosis of this syndrome can prevent diagnostic and treatment errors, such as unnecessary and invasive tests or biopsies, dentists' awareness plays a crucial role.

Therefore, this article reports the case of a patient with a clinical and radiographic appearance suggesting silent sinus syndrome. The patient was first referred to a dentist and treated with antibiotics due to a dental infection.

## 2 CASE REPORT

The patient was a 65‐year‐old male who complained of pain on the left side of his face and numbness in his upper lip on the same side. He was referred to the clinic of the Faculty of Dentistry, Mashhad University of Medical Sciences, Mashhad, Iran.

During the previous 2 weeks, the patient experienced swelling, redness, and inflammation on the left side of his face. The swelling decreased by taking antibiotics (amoxicillin and metronidazole every 8 h for 10 days), but the pain persisted, and numbness started in the upper lip.

About 40 years earlier, during the removal of the patient's teeth 25 and 26 on the left side, the maxillary sinus was opened, and the patient underwent surgery to close the sinus. However, since the sinus was not closed correctly, surgery was performed again through the nasal cavity on the same side. During these 40 years, the patient did not have any problems in this regard.

**FIGURE 1:**
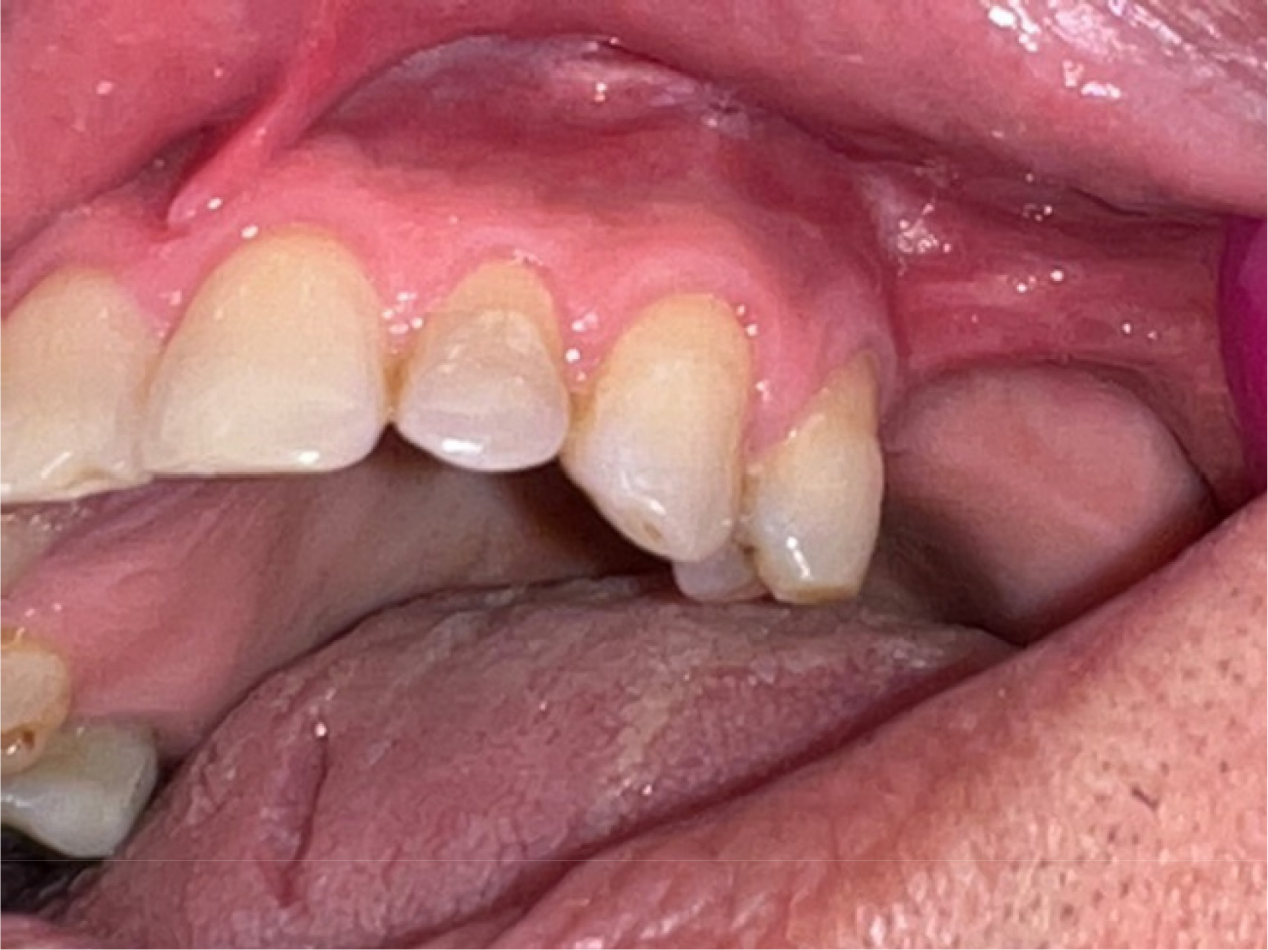
Intraoral view of the teeth and maxillary vestibule on the affected side.

According to the patient's medical history, he had had open heart surgery in the previous 2 years. He was then taking aspirin and atorvastatin. In the extraoral examination, there was no asymmetry or abnormal findings. In the intraoral examination, painless swelling was felt in the soft tissue of the vestibule depth from the mesial of tooth 23 to the mesial of the tooth 26, with a rubbery to firm consistency. In the examination, the teeth of the upper left quadrant were not sensitive to pressure. There was slight caries in the mesial of tooth 23, and the upper lip was unilaterally paresthetic on the affected side (Figure 1).

**FIGURE 2:**
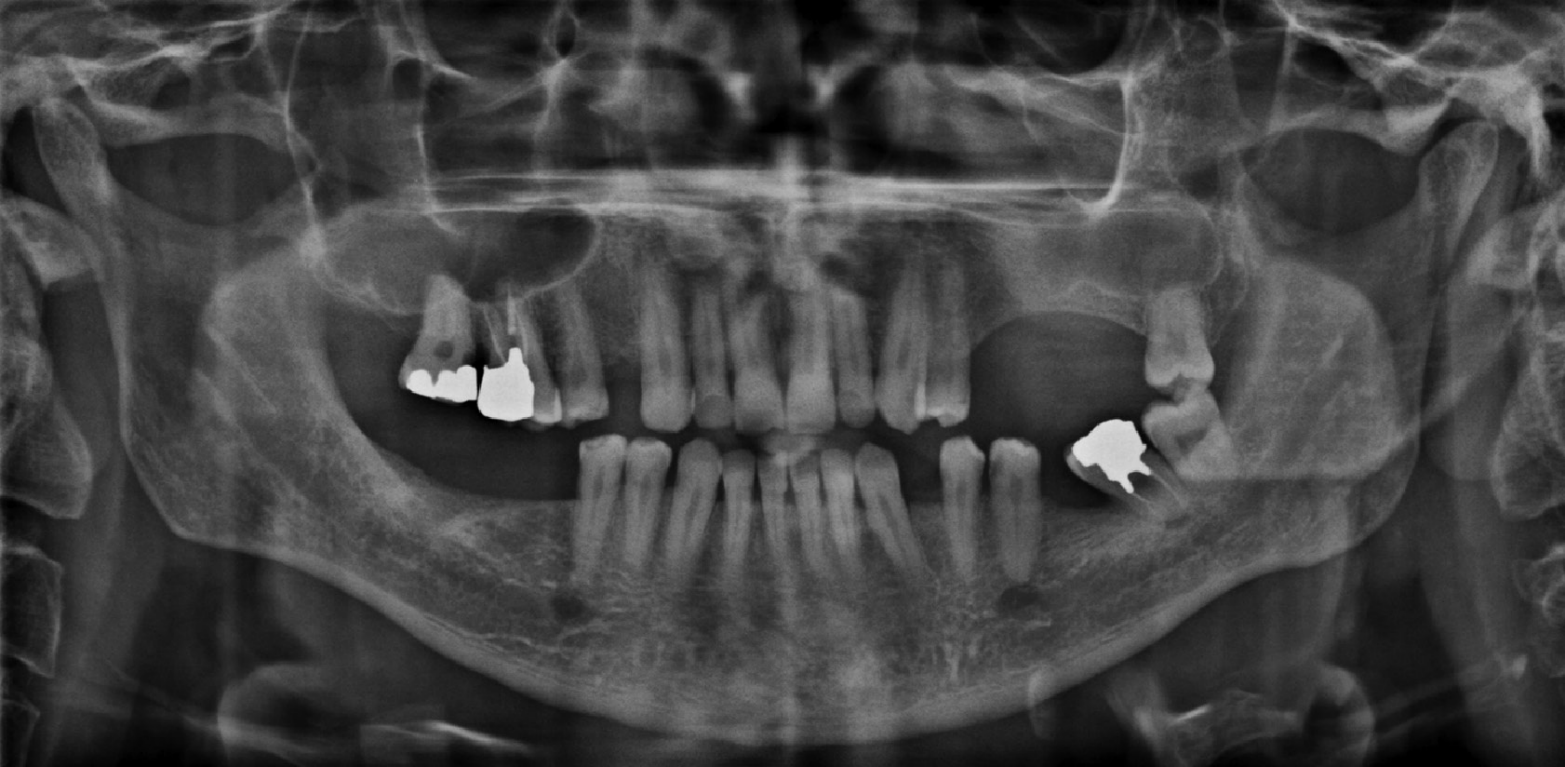
Panoramic view revealing the asymmetry of the maxillary sinus, sinus fullness, and weak integrity of the anterior wall of the left sinus.

An observation of the patient's panoramic view revealed the asymmetry of the maxillary sinus, per sinusitis, and the weak integrity of the anterior wall of the left sinus (Figure 2). According to the clinical examination and radiography, the patient was referred to a radiologist for a CBCT. This was to rule out the dental cause with the possible diagnosis of a sinus lesion, especially a malignant one.

**FIGURE 3:**
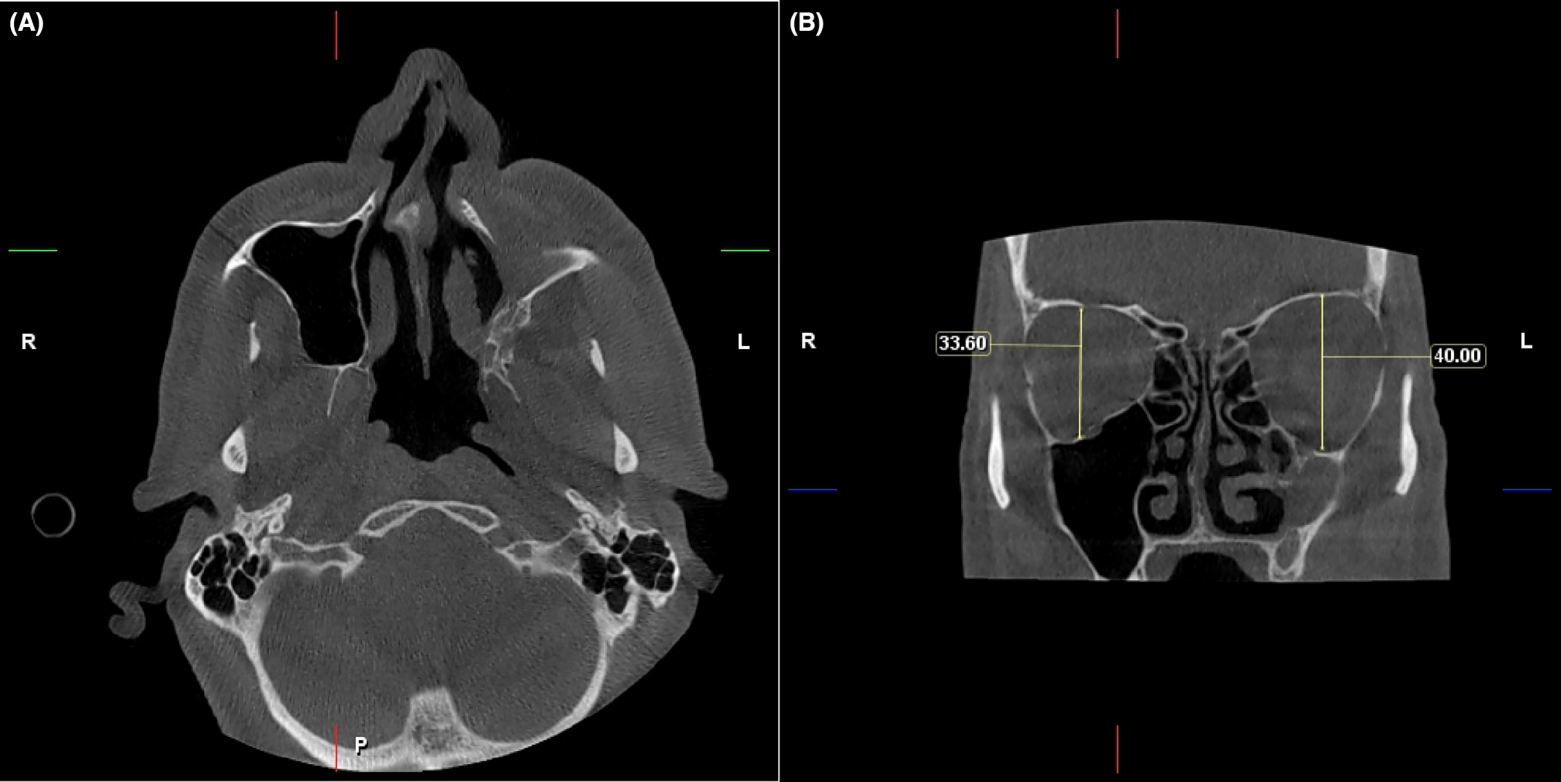
CBCT view. (A) The coronal plane shows the difference in the orbital cavity height and the asymmetry of the sinuses. The lower radiolucency of the left orbit indicates fat in this area. (B) The axial plane shows contraction and reduction of left sinus volume, opacity, and lack of integrity of its anterior wall.

The CBCT showed sinusitis and unilateral hypoplasia of the left maxillary resulting from the negative pressure caused by infundibulum obstruction. The lower wall of the orbital cavity was also pulling down on the same side, and there was a difference in the height of the orbital cavity on both sides (a height increases on the left side). There was also fat in the lower region of the orbital cavity on the left side, and the globus was pushed down (Figure 3).

**FIGURE 4:**
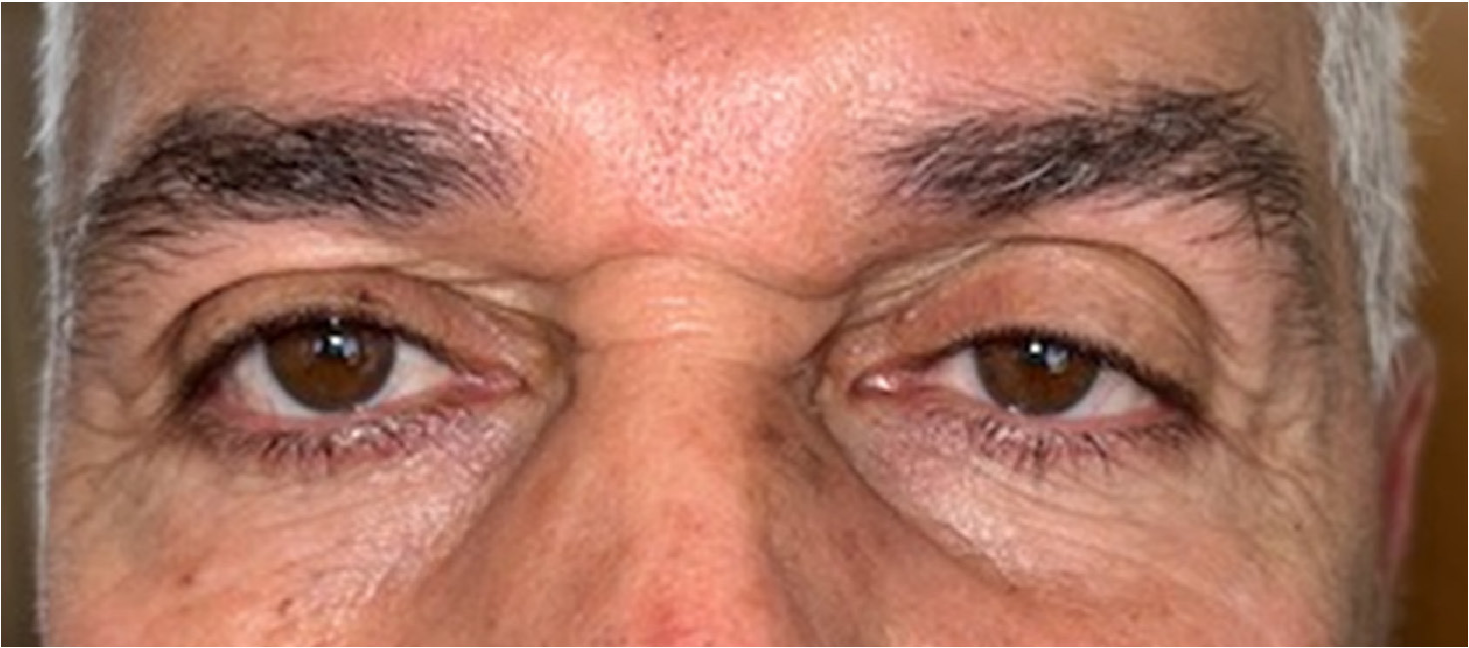
Clinical view of the patient's face showing eye asymmetry, enophthalmos, hypoglobus, and drooping of the upper eyelid in the left eye.

The radiographic interpretation and reexamination showed the asymmetry of the eyes and the enophthalmos and hypoglobus on the left eye. Based on these observations, the patient was diagnosed with “silent sinus syndrome” (Figure 4).

The patient was referred to an otolaryngologist for treatment and the opening of the infundibulum pathway. Due to his unwillingness to undergo surgery, the patient was treated only with antibiotics (amoxiclav, 32 tablets). At the two‐month follow‐up, the patient's pain and swelling improved, but the upper lip paresthesia still existed. Since there was no justification for the patient's persistent paresthesia, the patient was subjected to regular examinations.

## 3 DISCUSSION

The main symptoms of silent sinus syndrome include hypoglobus, spontaneous and progressive secondary enophthalmos, maxillary sinus hypoplasia, and orbital floor degeneration. Two underlying causes have been proposed for the occurrence of silent sinus syndrome.^4^ The first theory, known as “outlet obstruction”, claims that the acquired obstruction of the infundibulum disturbs the ventilation and accumulation of secretions in the maxillary sinus. The reason for the obstruction may be thickened mucus, lateral movement, or excessive movement of the infundibular inner wall, mucocele, or nasal polyps. Over time, the absorption of secretions leads to negative pressure in the sinus and the shrinking of its walls. The downward displacement of the orbital floor and its lack of support lead to hypoglobus and enophthalmos. These symptoms, including negative pressure in the sinus, are not common in chronic sinusitis.^3^, ^8^, ^9^

The second theory is the “mechanical theory”, postulating that the silent sinus syndrome is caused by negative pressure in the maxillary sinus, due to the contraction and hypotonia of the masticatory muscles, the aspiration of air from the closed space of the sinus, and the collapse of its walls.^10^

Eye trauma, chronic sinusitis, osteomyelitis, malignant infiltration, shrinkage and atrophy of eye contents (such as Wegener's granulomatosis, external radiation, and sclerosing tumor of the orbit), systemic inflammation (such as scleroderma), and pseudoenophthalmos are among the conditions that can result in hypoglobus symptoms and the development of enophthalmos. Unlike silent sinus syndrome, these conditions typically present signs and symptoms prior to the onset of hypoglobus and enophthalmos. Additionally, they can cause hypoglobus and less pronounced upper eyelid drooping.^11^

In previous studies, patients generally complained of facial asymmetry, enophthalmos, and hypoglobus without pain or diplopia.^3^, ^6^, ^10^ However, the main complaint of our patient at the time of the visit was pain on the left side of his face and paresthesia of the upper lip, with no signs of diplopia. In the initial examination, it was suggested that he had sinus malignancy in the first stage considering the pain and swelling for 2 weeks, the paresthesia of the upper lip, the absence of dental infection signs, the lack of improvement in the pain and paresthesia of the lip, and radiographic signs in the panoramic radiograph. Afterward, based on radiographic symptoms, the possibility of silent sinus syndrome was suggested. The timely diagnosis prevented unnecessary and invasive treatments, including biopsy.

In none of the previous studies, the main complaint of the patients was pain or paresthesia of the upper lip. They were mostly referred to dentists for asymmetry of the face and eyes. The cause of pain in our patient can be related to the blockage of the sinus tract, its lack of natural drainage, the retention of secretions, and its spread to the space around the sinus, which resulted in damage to the nerve endings, causing pain and paresthesia of the upper lip. Furthermore, the lack of integrity of the anterior wall of the sinus can be caused by the sinus closing surgery performed years earlier through the patient's left nasal cavity.

Before coming to the clinic, the patient had visited a dentist for swelling and pain and was suspicious of malignancy due to the lack of recovery and lip numbness. However, the dentist's awareness of and knowledge about this syndrome helped prevent unnecessary tests and aggressive treatment. It also calmed the patient's mind.

## AUTHOR CONTRIBUTIONS

**Samaneh Salari:** Methodology; supervision; writing – review and editing. **Najme Anbiaee:** Conceptualization. **Maryam Bozorgi Baee:** Data curation; investigation; resources; writing – original draft; writing – review and editing.

## CONFLICT OF INTEREST STATEMENT

There were no conflicts of interest to declare.

## CONSENT

Written informed consent was obtained from the patient to publish this report in accordance with the journal's patient consent policy.

## Data Availability

Data could be available by corresponding author with a reasonable request.

## Footnotes

* chronic contraction of maxillary sinus walls.

^†^
depression and downward movement of the eyeball.

^‡^
drooping of the upper eyelid.

^§^
asymmetric sunken eyes.
